# Neurocognitive impacts of arbovirus infections

**DOI:** 10.1186/s12974-020-01904-3

**Published:** 2020-08-10

**Authors:** Marion Clé, Patrick Eldin, Laurence Briant, Annie Lannuzel, Yannick Simonin, Philippe Van de Perre, André Cabié, Sara Salinas

**Affiliations:** 1 Pathogenesis and Control of Chronic Infections, INSERM, University of Montpellier, Etablissement Français du Sang, Montpellier, France; 2 Institute of Research in Infectiology of Montpellier, CNRS, University of Montpellier, Montpellier, France; 3 Neurology Unit, INSERM CIC 1424, Guadeloupe University Hospital, Université des Antilles, Pointe-à-Pitre, Guadeloupe France; 4 INSERM U1127, CNRS, UMR7225, Brain and Spine Institute, Sorbonne University Medical School, Paris, France; 5 Pathogenesis and Control of Chronic Infections, INSERM, University of Montpellier, Etablissement Français du Sang, CHU Montpellier, Montpellier, France; 6 INSERM CIC 1424, Infectious Disease and Tropical Medicine Unit, Martinique University Hospital, Université des Antilles EA4537, Martinique, France

**Keywords:** Arbovirus, Nervous system, Long-term sequelae, Cognition

## Abstract

Arthropod-borne viruses or arbovirus, are most commonly associated with acute infections, resulting on various symptoms ranging from mild fever to more severe disorders such as hemorrhagic fever. Moreover, some arboviral infections can be associated with important neuroinflammation that can trigger neurological disorders including encephalitis, paralysis, ophthalmological impairments, or developmental defects, which in some cases, can lead to long-term defects of the central nervous system (CNS). This is well illustrated in Zika virus-associated congenital brain malformations but also in West Nile virus-induced synaptic dysfunctions that can last well beyond infection and lead to cognitive deficits. Here, we summarize clinical and mechanistic data reporting on cognitive disturbances triggered by arboviral infections, which may highlight growing public health issues spanning the five continents.

## Background

Neurological sequelae, including cognitive deficits, are emerging as potential long-term impairments associated with some arboviral infections. Among emerging viruses, some arboviruses are able to reach the central nervous system (CNS) and cause neuropathology. Accumulating evidence highlighted by follow-up studies is now showing that neurological symptoms such as memory, behavior, and other psychomotor deficits are found in patients, months after the initial infection and decrease in some cases their quality of life. The aim of this review is to provide a comprehensive view of the neurological impairments found in some arbovirus infections that can have long-lasting effects, and to correlate these observations with molecular and cellular studies aiming to decipher the effects of CNS arbovirus interaction.

## Introduction

Accumulating evidence illustrate now the fact that neurotropic viruses have developed numerous strategies to invade the brain and, depending on the mode of entry, cellular tropism and mechanism of infection, trigger a wide range of neuronal symptoms, which can lead in some cases to severe cognitive impairments [1–3]. Once in the brain, altered neuronal homeostasis triggered by long-term inflammatory microenvironment and/or viral replication can have dramatic effects and lead to cognitive disorders [1, 3, 4]. For example, around 50% of human immunodeficiency virus (HIV)-infected patients are suffering from mild to severe neurological impairments in a syndrome called HIV-associated neurocognitive disorder (HAND) consisting of a range of cognitive deficits such as memory and attention disorders, motor and sensory impairment, mood and behavior changes and, in some extreme cases, dementia or HAD (HIV-associated dementia) [5]. HIV nervous system infection may also be linked to the etiology of some brain disorders such as amyotrophic lateral sclerosis [6] or Alzheimer’s disease (AD) [7]. Another example is found among members of the *Herpesviridae* family, such as cytomegalovirus (CMV), which can be associated with neurodevelopmental defects, as well as herpes simplex virus 1 (HSV-1), which induces latent infection in the nervous system and in some cases can trigger encephalitis when reactivated. Notably, both viruses were found to induce significant cognitive impairment in the general population [8] and were also proposed to be involved in the etiology of AD [9, 10]. “Hit and run” mechanisms leading to progressive neuronal pathology may be also considered, such as subacute sclerosing panencephalitis, a rare progressive neurological disease caused by complications associated with measles virus infection, which can have major impact including behavioral impairment cognitive decline and seizures [11].

**Fig. 1:**
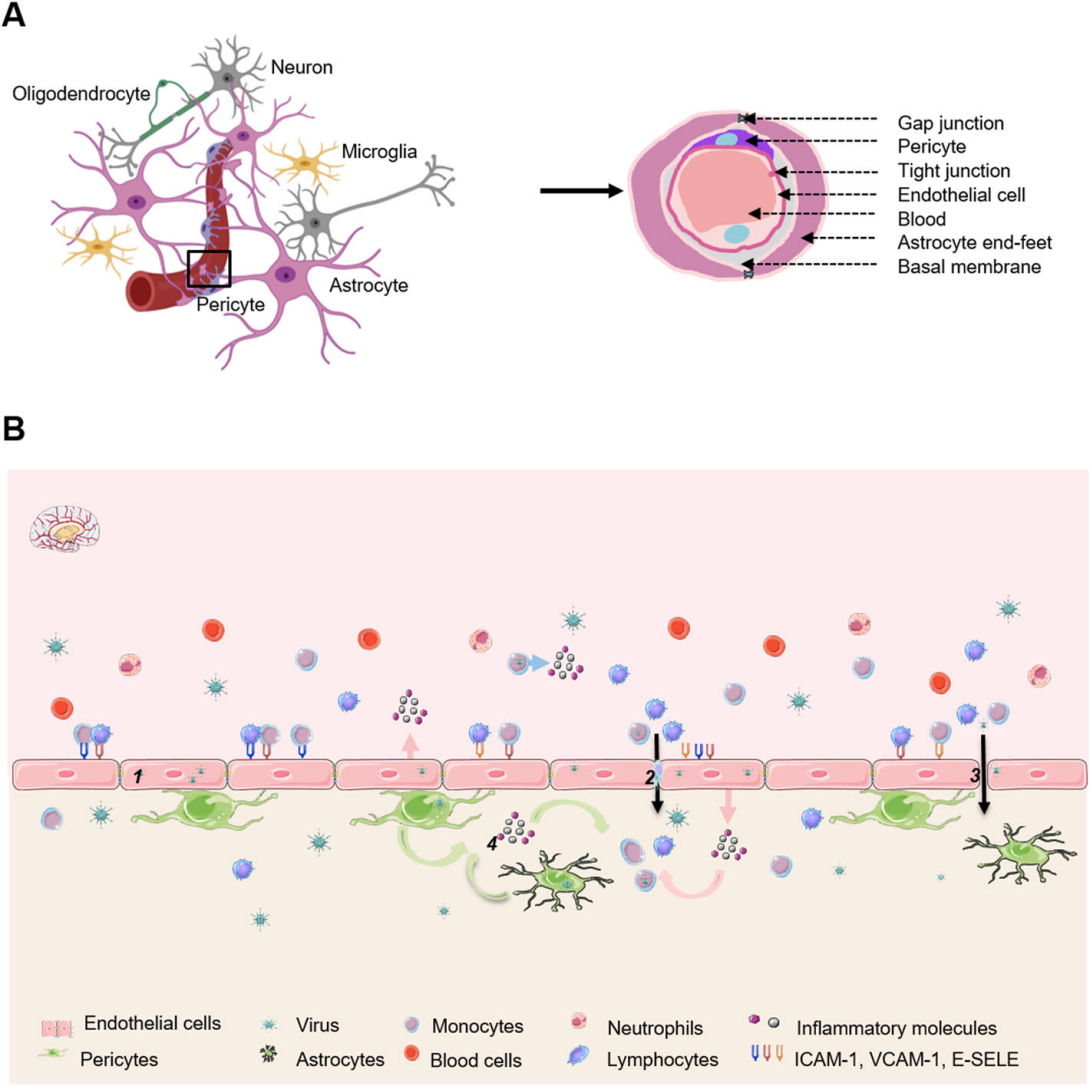
The NVU and pathways of CNS viral entry through the BBB. **a** The NVU is formed by astrocyte end feet, perivascular microglia, neurons, as well as brain pericytes, which are embedded in the basement membrane and envelop the endothelial cells lining cerebral capillaries. **b** Description of possible mechanisms of CNS virus through the BBB. (1) Direct infection of endothelial cells that release viruses in the brain. (2) Infection of monocytes infiltrating the CNS by the Trojan horse mechanism. (3) Infection of endothelial cells that disrupt the BBB by the release of inflammatory mediators. (4) CNS cells participate in the disruption of CNS homeostasis by producing inflammatory molecules and allowing the recruitment of immune cells. Images created with BioRender.com and SMART- Servier Médical ART

Due to its peculiar architecture, the central nervous system (CNS) is relatively protected from toxic and pathogenic factors that can be found in the blood, and in this light, it is considered as immune-privileged. This however does not exclude that some toxins, viruses, bacteria, or parasites can access this organ and cause mild to severe impairment. This can be done directly through pathogen-mediated effects on neurons, or indirectly through inflammation-associated mechanisms when glial cells are affected for instance. To reach the central and peripheral nervous systems (PNS), pathogens have been selected throughout evolution for their ability to interact with various barriers and machineries [12, 13]. Notably, the blood brain barrier (BBB) is a tight endothelium that physically separates systemic circulation from the parenchyma. It is formed by closely interacting cells, which form the neurovascular unit (NVU): vascular endothelial cells that actually form the barrier, pericytes, astrocytes, and neurons (Fig. 1) [14]. Endothelial cells are closely interacting through tight and adherens junctions (TJ and AJ respectively), which ensure the (relative) impermeability of the barrier, although anatomical sites, such as the choroid plexus (CP), are more vulnerable due to their loose inter-endothelial cell junctions. Selective passage nevertheless exists as small lipophilic molecules, cytokines, and cells of the immune system can cross the BBB using different mechanisms such as transcytosis through receptor-mediated endocytosis, transport with efflux/influx pumps, transcellular lipophilic pathways, and transcellular diapedesis [15]. Direct infection of endothelial cells can also provide viral access to both sides of the barrier. Numerous viruses have been shown to directly and/or indirectly (*e.g*., through infection of cells of the immune system, a mechanism called “the Trojan horse”) cross the BBB [16–19] (Fig. 1).

Many arboviruses (for arthropod-borne viruses), the majority of which are responsible for acute infections, can also access the CNS and infect a variety of cell types [20]. Because encephalitic arboviruses encompass numerous viruses belonging to various families, one cannot tend to generalize the (neuro) pathologies associated with these infections. However, these viruses are in the vast majority ribonucleic acid (RNA) viruses and transmitted by vectors such as mosquitoes, ticks, and sandflies during blood meals to a range of host including humans [21]. The replication cycle of arboviruses generally occurs in wild hosts such as birds and mammals. Vectors are then responsible for spreading infection among hosts in what is called an enzootic cycle [22]. In some situations, vectors can transmit viruses to animals that are not the natural host (*i.e*., do not replicate the virus), which are called accidental or dead-end hosts. This is the case for example for horses and humans following West Nile virus (WNV) or Usutu virus (USUV) infections [23, 24]. Some arboviruses such as dengue virus (DENV), Zika virus (ZIKV), and chikungunya virus (CHIKV) are less relying on viral amplification in wild animals and can be transmitted to humans during an urban cycle, and therefore are found associated with major epidemic outbreaks [21, 22]. Arboviral CNS infections can happen with a wide range of arboviruses including WNV, ZIKV, CHIKV, USUV, and Japanese encephalitis virus (JEV) among others, and cause diseases such as meningitis, encephalitis, meningoencephalitis, myelitis, and acute paralysis [20, 25]. Because arboviral infections were mostly seen as acute, neurological symptoms, with the exception of neurodevelopmental impairment, were also largely reported on a short-time range. The recent ZIKV epidemic and the congenital syndromes associated (*e.g*., microcephaly [26]) suggest however that arboviral infections may have long-lasting effects on the nervous system. We review here the clinical and basic studies aiming to characterize the long-term effects of arboviral infections, in particular regarding cognitive performance.

### Acute and long-term cognitive deficits in arbovirus infections—a clinical perspective

#### Arboviral infections and symptoms

Clinically, arboviral infections are acute (viremia is typically few days long) and most frequently asymptomatic (60–80% of patients) or trigger flu-like symptoms resulting in febrile state, mild to important fever, and, depending on the virus, can be accompanied by rash, conjunctivitis, myalgia, and cephalic pains [22]. In rare cases, severe symptoms such as hemorrhagic fever (*e.g*., for DENV and Crimean-Congo hemorrhagic fever virus (CCHMV) infections) or neuropathology (*e.g*., for WNV, Toscana virus, JEV, USUV, ZIKV…) can be found associated with arboviral infections. During epidemics however, even “moderate” symptoms (which still necessitate hospitalization) can be of a great burden, as it was well illustrated during the 2005–2006 CHIKV epidemic in the French island of La Réunion, which affected up to a third of the population and had a huge cost for the society [27, 28], or with DENV, which is present in 130 countries and put up to 2.5 billion people at risk each year [29]. This is particularly relevant for endemic arboviruses, which should be closely considered by clinicians [30], but given the current globalization of travelers and merchandises, as well as climate change, it is more than likely than emerging and remerging viruses will found “new” or naïve territories, similarly to what happened for ZIKV in South America or CHIKV in America [31, 32].

Moreover, long-term sequelae (lasting from weeks to years) can be found occurring after arboviral infections in some cases [33] (Table 1). CHIKV infections have been linked to lasting arthralgia and arthritis, among other symptoms, which directly impact the quality of life [68, 80, 81]. Ocular complications, reported for several arboviruses including WNV, CHIKV, DENV, and ZIKV among others, are often associated with long-term impairment [33, 43]. For instance, in ZIKV-infected adult patients suffering from visual impairment in the acute phase of infection, follow-up studies showed partial recovery as permanent lesions due to ZIKV infection may be likely to persist [82, 83]. Recent data also point that ZIKV can persist in various body fluids for weeks to months, switching the paradigm of seeing (some) arbovirus infections as acute, towards more persistent or long-term infections [46, 84].

**Table 1:** Neurological disorders associated with arboviral infections

Virus	Neurological disorders	Long-term sequelae in children and in adults	References
WNV	Encephalitis, meningitis, meningoencephalitis, and acute flaccid paralysis	Neurological sequelae, confusion, seizure, memory impairment, speech disability, depression, and ocular complications	[34–42]
DENV	Encephalopathy, meningitis, stroke, cerebellar syndrome, myelitis, and guillain-barré syndrome	Neurological sequelae, mood-, personality-, behavior- disorders, and ocular complications	[29, 43–45]
ZIKV	Meningoencephalitis, guillain-barré syndrome, microcephaly, and congenital Zika syndrome	Mental retardation, seizure, epileptic behavior, communication-, social cognition-, and mobility- abnormalities, autism spectrum disorder, intellectual disability, memory and learning deficits, and ocular pathology	[46–62]
JEV	Encephalitis, aseptic meningitis and acute flaccid paralysis	Motor and language deficit, learning difficulties, behavioral problems, mental retardation, neurological-, neuropsychiatric-, and cognitive sequelae	[63–67]
CHIKV	Encephalitis, myelopathy, neuropathy, myeloneuropathy, myopathy, and paralysis	Arthralgia, arthritis, neurological sequelae, cognitive disturbance, and ocular complications	[68–73]
TBEV	Encephalitis, meningitis, meningoencephalitis, and myelitis	Cognitive sequelae, behavior-, memory-, and language- dysfunctions	[74–77]
EEEV, WEEV	Encephalitis	Seizures, cognitive defects, psychiatric illness, motor dysfunction, behavioral impairments, and intellectual impairment	[78, 79]

#### Arboviral infections and neuroinflammation

More worrying however, are the neurological impairments that can be directly triggered by arboviruses, both from congenital and adult infections (Table 1). CNS pathology during arbovirus infection can be due to direct neuronal infection, but also from indirect effects due to global neuroinflammation and post-infectious mechanisms that can occur in distinct anatomical regions. Regarding the brain, while inflammation isolated to the meninges triggers meningitis, viral replication in the brain parenchyma results in encephalitis. Aseptic meningitis is classically defined as non-bacterial inflammation of the tissues lining the brain. Any inflammation or pathology that also involves the parenchyma is referred to as meningoencephalitis. “Neurotropic” (*i.e*., able to reach the CNS) arboviruses are classically associated with encephalitis [63, 78, 85–87] but can also cause meningitis, for instance in infections by St. Louis encephalitis virus (SLEV) [88], tick-borne encephalitis virus (TBEV) [89], DENV [90], WNV [91], CHIKV [92], ZIKV, Powassan virus (POWV), and Eastern Equine encephalitis virus (EEEV) [93]. During Toscana virus meningitis, serum levels of IFN-α, IP-10, and eotaxin are significantly increased in the acute phase of infection in comparison with healthy controls [94], which could participate to an ensuing CNS infiltration of neutrophils, monocytes, and antiviral CD8^+^ lymphocytes. In patients suffering from encephalitis or meningoencephalitis due to WNV, perivascular and meningeal inflammation is found, both in the brain and in the spinal cord, which is associated with seizures and paralysis [20]. This illustrates the potential neuroinflammation found in some arboviral infections, which can cause acute and long-term neurological impairments [95].

#### Congenital and pediatric arboviral infections and neurodevelopment

Congenital arbovirus infections have been particularly reported for ZIKV, mostly due to the extent of the epidemic [47]. In this setting, microcephaly (which results in a decrease in head circumference and brain growth) and other cerebral malformations (called altogether congenital Zika syndrome or CZS) have been consistently reported throughout the American continent, but also elsewhere in the globe [47–51]. Moreover, retrospective studies in past epidemics, such as the one from French Polynesia, showed indeed that microcephaly and cerebral malformations were associated with ZIKV infection [52, 53]. Lissencephaly (malformation in the cortical structures of the brain) has also been reported in ZIKV-infected infants, which could result in mental retardation in affected children [54]. Importantly, there are now follow-up studies showing the effects from ZIKV-associated brain malformations or subtler damages. Such studies in infants with CZS confirmed strong neurological disabilities, in particular ocular and motor impairment, as well as epileptic manifestation [48, 55–60]. A study assessed over 1400 children of at least 1 year of age born from ZIKV-infected mothers and showed important rates of neurodevelopmental defects (up to 14% with seizure or, neurodevelopmental delays) [61]. Report on more than 200 children born from ZIKV-infected mothers in the region of Rio de Janeiro also showed important neurodevelopmental, including cognitive, impairment in around 30% of patients 2 years after birth [62]. The microcephaly status was shown to be altered in some children, either resolving or appearing [62]. Appearance or further development of neurological impairments can be also occur, consistent with observations that ZIKV can persist in infants after birth for a period of months (which is adding from the several weeks of ZIKV infection *in utero*) [54].

However, other arboviruses have been reported to be associated with pediatric and/or congenital neuronal disorders, in particular DENV [44], CHIKV [80, 69], and rarely WNV [96]. CHIKV infection is now well established as triggering serious neurological sequelae, particularly in children [70, 71]. Vertical transmission has been documented in several studies and highlights the risk of congenital infection associated with this virus [71–73]. Neurocognitive outcome of a La Réunion cohort of 33 CHIKV-perinatal exposed infants showed neurodevelopmental delays in around 50% of children compared to controls [69]. Cases of encephalopathy, microcephaly, and cerebral palsy were also described, some of them not detectable at birth and developing afterward, similarly to ZIKV-infected children [69]. Moreover, in a cohort of 87 children CHIKV^+^ (CHIKV RNA found in cerebrospinal fluid (CSF)) (55 infants less than 1 year old with an acute infectious syndrome and 32 children 2 to 10 years old with a convulsive attack or encephalopathy), evaluated 3.5 to 4.5 years after acute chikungunya, 20% of children presented developmental delays including cognitive impairment (our unpublished data).

Infants and children are also particularly at risk for DENV infection, potentially with severe forms [29]. Among these disorders, severe encephalopathy can be found (e.g. [45, 90],) but no long-term follow-up that suggests potential cognitive sequelae was reported as far as we know. This would definitely need to be addressed as DENV affects hundreds of thousand persons each year. Noteworthy, Rift valley fever virus (RVFV) is associated with vertical transmission and fetal demises in animals and with neurological and ocular impairment in humans [97]. Its epidemic potential is strongly considered as the WHO classified it as “severe emerging disease with potential to generate a public health emergency, and for which no, or insufficient, preventive and curative solutions exist” and is a reported as a category A priority pathogen [98]. Rare human vertical transmission have been reported, one of which resulted in infant death within a week [99]. Whether this is due to intrinsic properties of the virus (see below) or of poor surveillance diagnosis needs to be characterized. WNV vertical transmission does not seem a common feature but infections in children occur regularly [100]. These infections can give rise to meningitis, encephalitis, and acute flaccid paralysis, the latter of which can cause long-lasting disabilities but, curiously, neuroinvasive disease in children is less frequently found than in adults [100]. TBEV can also affect children but, similarly to WNV, infections are in general milder than in adults [74]. However, European cohort’s studies in children demonstrate important rate of neurological and cognitive sequelae after TBEV infection [74, 75]. In the same light, other arboviral infections such as JEV or La Cross Virus (LACV) in children may be associated in some cases with neurological and cognitive sequelae [63, 87]. In a follow-up study over 2 years post-JEV infection in children showed mental retardation in over 20% of patients [64].

Altogether, these observations highlight the existence of potential risks associated with arboviral infections during pregnancy or in early life for child neuronal development [101]. In this context, it was also discussed that the neurodevelopmental defects associated with ZIKV infection *in utero* could potentially favor autism spectrum disorder [102]. Moreover, generally infectious encephalopathies in children represent important risks to develop neurological and cognitive sequelae [103], suggesting that arboviral infections in children may have severe consequences for the neuronal health of the individuals.

#### Arboviral infections and acute and long-term cognitive impairment in adults

Encephalitis, meningitis, and other neuronal complications following arboviral infections are also found in adults, with sometimes long-term cognitive impacts. In particular, neurological sequelae have been well described in WNV adult patients [34–39]. For instance, in a 1-year post-infection follow-up study of WNV patients who were diagnosed neuroinvasive disease, several neurological sequelae were reported including memory impairment, speech disability, and depression [34]. Similarly, mental health and social functioning were altered in some Canadian neuro-WNV patients in a 2-year follow-up study [40] and in another 2 to 4-year follow-up study [41]. Moreover, in 1–3 and 8–11 year follow-up study of the Houston West Nile Cohort, new neurological symptoms developed in some patients, highlighting the need to closely monitor post-encephalitic patients [42]. Similarly, the highly neurotropic JEV when infecting adults also led to significant increase in cognitive and behavior impairment in patients several years post-infection [65]. A prospective study of over 1300 Indian adult patients initially diagnosed with JEV infection highlighted the potential risk to develop neurological and cognitive sequelae after JEV infection: the authors showed in these patients of four epidemics between 1978 and 1989, neuropsychiatric and neurological sequelae at the time of discharge [66]. Some of these patients were then enrolled in follow-up studies (up to 14 years) that showed important neuronal sequelae including corticospinal impairment such as hyperkinetic movement and dystonia and seizures [67]. Psychiatric and psychological disturbances were also observed as long lasting: some patients were reported even though some recovery occurred [67]. Intellectual disability, memory, and learning deficits were also seen in a small proportion of patients [67].

Cognitive sequelae are also clearly associated with TBEV infection in adults; here, it can sometime be associated with neurological symptoms such as meningitis, encephalitis, and meningoencephalitis. Long-term follow-up studies highlighted the risk for cognitive sequelae: for instance, in a study following 36 Polish farmers diagnosed with TBEV infection during the 1994–2001 period, the authors reported cognitive impairment related to the pre-dementia stage of AD or memory and language dysfunction [76]. Similarly, in a Scandinavian adult cohort of 96 TBE patients, cognitive disorders such as changes in behavior and learning disabilities were reported in a follow-up study 2 to 15 years post-infection [77]. CHIKV can also lead to neurological disorders in adults, mainly encephalitis but also acute paralysis [71]. Importantly, neurological sequelae, including cognitive disturbance were found in adults who developed at the time of infection CNS disorders in a 2-year follow-up examination [85]. Albeit neurological complications associated with ZIKV infections have been mainly described in pediatric cases, adults can also develop brain disorders [104]. For instance, behavioral changes were observed up to 15 weeks in a ZIKV-infected teenager [105].

Exposure to other emerging neurotropic arboviruses can have long-term deleterious effect on mental health as illustrated by a case of a 73-year-old patient infected with California Serogroup Virus who developed post-encephalitic dementia and was transfered to a nursing home [106]. More than 6 months post-infection, the patient still scored low on a mental state examination (11/30) [106]. Evenly worrisome, a follow-up study concerning Murray Valley encephalitis virus showed that even in patients discharged without evident sequelae, long-term cognitive disturbance such as depression and cognitive dysfunction were reported [107]. Alphaviruses have also been associated with neuronal impairment with long-term effects. Eastern, Western, and Venezuelan Equine encephalitis virus (EEEV, WEEV, VEEV) human infections have also been described, some of which led to cognitive sequelae [78, 108, 109]. Outbreaks of WEEV in the USA and in Canada in the 40–50s were proposed to be associated with important neuronal sequelae including behavioral impairments [79, 110]. Follow-up studies of later WEEV outbreaks showed that some children and adult suffered from intellectual impairment [108].

### Impairment of neuronal functions by arboviruses—molecular and cellular mechanisms

Cognitive deficits can stem from different mechanisms, whether from impairment of neuronal development, neuronal dysfunction from a direct impairment of neuronal homeostasis (*e.g*., direct neuronal infection and function perturbation), or through indirect effects mediated by inflammatory molecules released by infected glia or immune cells. To understand the interaction and its effects between arboviruses and the nervous system, numerous studies aimed at characterizing the effect of infections on brain development, CNS entry mechanisms, and the cellular and molecular effects associated with brain infection and neuroinflammation.

#### Mechanisms of neuronal development modulation by arboviruses

Studies in animal models clearly showed that *in utero* or early post-natal ZIKV infections may have deleterious effect on neurodevelopment and trigger cognitive disorders in growing mice [111–113]. Importantly, ZIKV has been shown to potently cross the placenta using several mechanisms including infection of trophoblasts in the placenta, which further allow spread in the fetal nervous system [114].

Further studies showed that other arboviruses can infect human placenta and could be associated with fetal demise in animal models: a study showed that in human placental explants ZIKV, WNV, Mayaro virus (MAYV), and POWV, but not CHIKV, were able to replicate within different components of the tissue [115]. Similarly, RVFV can cross rat placenta and trigger deleterious effects on developing embryos [116]. Importantly, high viral load was detected in the pup brains [99]. Moreover, human placenta explants were also found permissive to RVFV replication [116], confirming the observations of human vertical transmission [99].

Numerous studies showed potent ZIKV infection of neuroprecursors and important neurodevelopmental defects (*e.g*. [117],). This infection is deleterious for the subsequent neuronal differentiation and the establishment of synapses [118]. Globally, *in utero* ZIKV infection led to cortical thinning resulting from neuronal growth defect and neuronal death [119]. This was accompanied by a reduction in neuronal network, suggesting important neurodevelopmental defects in developping animals, consistent with cognitive disturbances observed [112, 120]. Cell cycle dysfunction has been linked with ZIKV infection of neuroprogenitors [119, 121–123], which is likely to explain effects in neuronal differentiation and apoptosis as progenitor division is strictly necessary for neurogenesis. Due to extensive research following ZIKV epidemic and with the above observations in mind, researchers and clinicians agree around a consensus regarding the cause of ZIKV-mediated microcephaly: potent infection of neuroprogenitors, coupled with cell death, differentiation, and neuronal network impairment are believed to be responsible for this congenital brain development disorder [124]. Regarding effects on the developing brain, much less data is available for other arboviruses. Similarly, JEV impairs subventricular zone neuroprogenitor proliferation and cell cycle progression through modulation of checkpoint proteins in wild-type (WT) mouse pups and in vitro [125]. Surprisingly, albeit clear neurodevelopmental and cognitive impairment have been shown in CHIKV-infected children [69], very little information is found on the physiopathology associated with these effects, albeit it was hypothesized that CHIKV could target neural progenitors and affecting neurogenesis [126]. Even though rare cases of congenital infections have been reported with WNV, *in utero* infection in a mouse model showed effect on brain development [115]. These studies corroborate observations in arboviral congenital or perinatal infections and their effect on neurodevelopment and cognitive functions.

#### Mechanisms of arbovirus brain access

To reach the adult CNS, neurotropic arboviruses use different mechanisms at the BBB such as direct viral infection of brain vascular endothelial cells or using cells of the immune system (“Trojan horse” pathway) [127]. It was proposed that WNV enters the CNS by infecting monocytes, dendritic cells, or macrophages that naturally cross the BBB [128, 129]. Similarly, JEV and DENV have been shown to use such pathways [127]. Direct infection of endothelial cells may have different outcomes for viral access: some viruses such as ZIKV may not have important effect on BBB integrity but can be released through the basolateral compartment and reach the CNS [130]. Others may have more potent effects on BBB permeability through production of inflammatory cytokines, which can modulate BBB integrity. For instance, WNV by infecting directly brain vascular endothelial cells will lead to the production of inflammatory molecules that will disrupt BBB integrity and further allow virus CNS access through the Toll-like receptor (TLR)-3 response and tumor necrosis factors alpha (TNF-α) secretion, resulting in a transient change BBB permeability [131]. DENV can directly infect human microvascular endothelial cells and induce cell apoptosis [132]. Moreover, modulation of TJ and AJ protein expression by arboviruses can also occur and increase viral and immune cell CNS access by paracellular pathway [133]. Studies also showed that neurotropic arboviruses can upregulate cell adhesion molecules in brain vascular endothelial cells, which in turn favor leukocyte recruitment and CNS invasion [134, 135]. Furthermore, some arboviruses can invade the brain at the blood-CSF interface [136–138]. Some flaviviruses are able to use the olfactory pathway to enter the CNS such as Murray Valley encephalitis virus and Saint Louis encephalitis virus. Others will use retrograde axonal transport to access it using peripheral nerves such as WNV [139–141].

#### Arbovirus and interaction with the neurovascular unit

Once in the brain, neurotropic arboviruses can also infect others types of NVU cells such as pericytes, astrocytes, neurons, and microglia and lead to general neuroinflammation and BBB impairment [14, 142]. Because astrocytes are mediators of neuroinflammation, these infections may further impair BBB homeostasis [143] and disrupt neuronal viability and induce cognitive dysfunction [144, 145]. Astrocytes can be infected by numerous arboviruses such as TBEV, WNV, ZIKV, and JEV [146]. In this context, WNV-infected astrocytes have been shown to secrete various inflammatory cytokines and matrix metalloproteinase (MMP), which will lead to BBB disruption [147, 148]. ZIKV-infected human astrocytes produce pro-inflammatory cytokines that induce neuroinflammation [149] and in mouse embryo brain, astrocytes-infected ZIKV induce progressive astrogliosis, which disrupts BBB properties and function [119]. Similarly, astrocytes infected with JEV lead to secretion of MMP, interleukin (IL)-6, and vascular endothelial growth factor (VEGF) and led to BBB destabilization [150]. JEV-infected astrocytes also led to an increase of the chemokine (C-X-C motif) ligand 10 (CXCL10) production, which modulates the migration of natural killer (NK) cells and monocytes into the CNS [151]. Moreover, arbovirus infection of astrocytes often results in the production of cytokines such as IL-6, TNF-α, or IL1-β, which have been shown to modulate BBB permeability by several mechanisms, including downregulation or relocalization of junction proteins such as occludin and zona occludens (ZO)-1 [147, 152–154]. The NVU is also composed of pericytes, which support BBB homeostasis and function, and are emerging as key regulators in neuroinflammation [155, 156]. JEV, WNV, and ZIKV have been shown to target pericytes. JEV-infected pericytes in turn induce a degradation of TJ proteins such as zonula occludens (ZO)-1 protein and an upregulation of ubiquitin E3. Moreover, JEV-infected pericytes produce IL6, which disrupted the integrity of endothelial barrier [157, 158]. Noteworthy, the BBB is also impaired during congenital infection as *in utero* ZIKV infection in mice led to abnormal vasculature, which triggered BBB leakage [119].

Arboviral interaction/modulation with the NVU may have important and potentially long-term effects as BBB and vasculature impairment are associated with cognitive disturbance either in an acute fashion, or in a long-term manner, for instance in aging [14]. Neurodegenerative disorders such as AD are multifactorial. In this context, vascular impairment has been proposed to contribute to the etiology of such diseases [14, 159]. BBB dysfunction, coupled to pericyte degeneration may provoke toxic accumulation in the brain and neuronal dysfunction [14]. Similarly, repeated BBB injuries have been proposed to trigger neurodegeneration and neurocognitive dysfunction [160, 161].

#### Arbovirus and modulation of synaptic function

 Some studies aimed to address synaptic homeostasis both during and after arboviral infections using animal models, which allow testing for cognitive disturbance. Cognition is a complex and multifactorial mechanism, whereupon synaptic plasticity plays a central role. This is occurring early in development when synaptogenesis and maintenance are taking place, and during adulthood with a balanced regulation of synapse formation and removal, collectively termed synaptic plasticity [162]. To these mechanisms can be added adult neurogenesis, in particular in the hippocampus, where new neurons are constantly generated and integrated in existing circuits to modulate learning and memory mechanisms [163]. In the context of arboviral infections, mature neurons can be directly infected by some arboviruses including ZIKV, WNV, TBEV, and JEV among others [139, 164–166]. For instance, ZIKV has been shown to replicate in mature neurons ex vivo in human cortex and in vivo in mouse models [165]. However, synaptic plasticity does not only involve only neurons but also interactions with glial cells such as astrocytes and microglia [162]. Microglia are key regulator of neurodevelopment, neuroinflammation, and BBB integrity [167]. An important mechanism involved in cognition is microglia-dependent synaptic removal, which allows synapse remodeling called synapse pruning [167]. This mechanism, when exacerbated, can cause cognitive dysfunction [168, 169]. In an infection paradigm, activated microglia can be directly associated with neurodegeneration and cognitive defects [170].

**Fig. 2:**
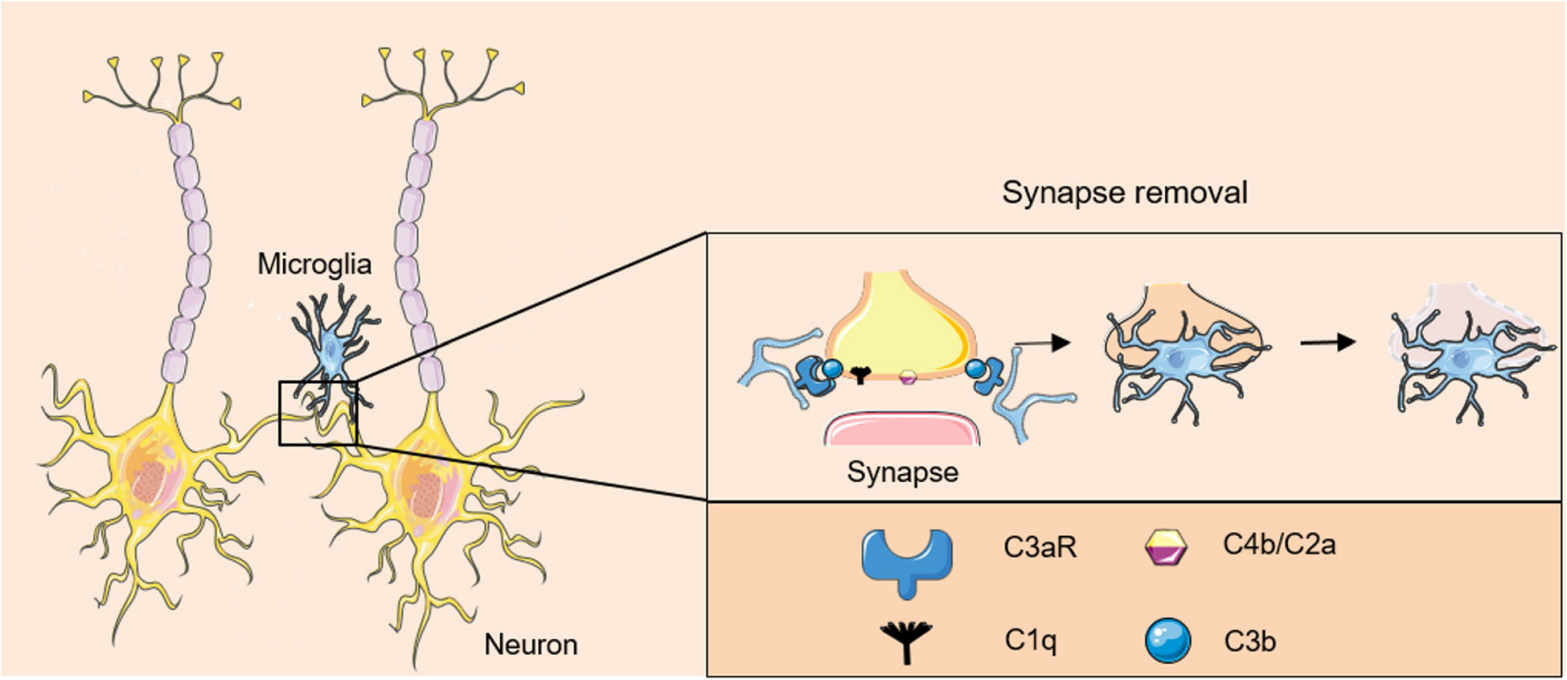
Elimination of synapses by microglia via the complement pathway. Activation of microglial complement receptors during arboviral infection triggers the phagocytosis of synapses. Complement components C1q, C4b/C2a, and the C3 fragment (C3b) tag synapses. Microglial cells bind C3b through their CR3 receptors and partially phagocyte tagged synapses, resulting in selective synapse elimination. Images created with SMART- Servier Médical ART

Microglia infection/modulation has been described in several cases: ZIKV can lead to a potent inflammatory response in human fetal brain microglia [171]. JEV and DENV have also been shown to target microglia, which induce inflammatory environment [172, 173]. Using the rat as an animal model, authors showed that JEV led to behavioral changes including memory impairment that could be correlated with a decrease in the synaptic protein choline acetyl transferase (ChAT), a marker of dopaminergic neuron function [174]. Microglia-dependent synaptic pruning has been proposed as a potential mechanism underlying neurocognitive impairment in patients recovering from WNV neuroinvasive disease. Indeed, observations in mice infected with a WNV mutant NS5 (E218A) suggest that the complement components (C3 and C3aR) mediate presynaptic terminal loss in the hippocampi of mice that exhibit spatial learning defects during recovery from neuroinvasive disease [175] (Fig. 2). Microglia and recognition of C3 cleavage products by the complement receptor C3aR were shown responsible for this process [175]. Indeed, disease-recovered animals (who survived the neuroinvasive disease) showed learning deficits that were mirrored by the presence of engulfing microglia at synapses in the hippocampus. In particular, synaptic terminal of the C3 regions were reduced both in animal models and in biopsies of human patients [175]. Furthermore, in adult mice that recovered from WNV and ZIKV infections, activated microglia through the release of interferon (IFN)-γ from infiltrating T cells were responsible of synaptic removal without repair in the case of WNV, and with associated neuronal apoptosis for ZIKV [176, 177]. It was proposed that CD8^+^ T cells, through the release of inflammatory molecules and microglia activation and subsequent synaptic removal, were participating in the establishment of post-infection cognitive sequelae [176]. In another study, ZIKV replication in the hippocampus was shown to lead to the inhibition of long-term potential, a key mechanism regulating cognitive process such as memory [165]. The authors demonstrated the presence of activated microglia in close proximity to synaptic terminals and memory impairment in infected mice [165].

Some studies also point towards the modulation of adult neurogenesis by some arboviruses. During the acute phase of WNV infection, myeloid cell-derived IL1 alters the proliferation and differentiation fates of neural progenitor cells, leading to a shift from neurogenesis to astrogenesis [178]. Data indicate that the combinatorial effect of synapse loss and reduced neurogenesis can negatively impact hippocampal spatial learning and memory long beyond the initial episode of infection via a shift in sources of cytokines cells [178]. ZIKV-infection on human astrocytes led to disruption of pathways and cellular protein levels involved in synaptogenesis axonal guidance signaling [179]. This could also participate to ZIKV-induced impairment of neuronal circuits and network development [179]. ZIKV also targets adult neuroprogenitors and was shown to alter hippocampal neurogenesis by inhibiting neuroprogenitor development [180]. The modulation of adult neurogenesis, for instance during inflammatory process, has been clearly linked to cognitive disturbances [181]. Whether modulation of adult neurogenesis is a process commonly find in arboviral brain infection still remained to be addressed but could contribute, partly, to acute and potentially long-term cognitive impairment.

#### Modulation of the neuro-epigenome by arboviruses

Another crucial mechanism in brain development and maintenance and associated cognitive functions, are the regulation of neuroepigenetic modifications [182, 183]. The ability of some arboviruses to establish productive infection in brain cells, to evade their antiviral responses, and to impair neurodevelopment and neuronal homeostasis relies on their intrinsic capacity to evolve complex and multifaceted modes of interactions with their various cellular hosts. Among these modes of virus-host relationship, manipulation of the viral and cellular epigenome and epitranscriptome during infection has recently appeared as a complex and dynamic landscape of deoxyribonucleic acid (DNA) and RNA “decorations” that can be hijacked by arboviruses to promote their replication and could also participate to neuropathogenesis and long-term defects. ZIKV has been shown to alter the DNA methylome of neural progenitors, astrocytes, and differentiated neurons at genes that have been implicated in the pathogenesis of a number of brain disorders, most prominently mental retardation, autism, and schizophrenia [184]. The virus-mediated alteration of gene and gene products of DNA modifiers involved in the control of the dynamic cycle of methylation/demethylation of cytosine is currently investigated [185, 186]. These studies might reveal how arboviruses may take over the dynamic C/5mC/5hmC switching process known to be critical for normal brain development and neuronal functions [187]. Manipulation of the viral and cellular epitranscriptome by positive-sense RNA viruses during infection may also control cellular and viral RNA stability and translatability drive subversion of the host and evasion of cellular surveillance systems [188, 189], preferentially promoting viral RNA translation, and eventually facilitating viral production. RNA modifications, including several nucleoside methylations, have long been known to be essential in the proper function of transfer (t)RNA and ribosomal (r)RNA. Recent analysis of RNA post-transcriptional modifications (PTMs) in the context of arboviral infections has underscored their complex and dynamic nature on either cellular or viral RNAs. In infected target cells, erasure of N6-methyladenosine (m6A), the most abundant modification of messenger (m)RNA, was shown to be profitable for particle production for several flaviviruses including hepatitis C virus (HCV) and ZIKV [190, 191]. In contrast, Flaviviridae infection has recently been shown to increase the expression of specific cellular factors (such as RIOK3 and CIRBP) that turned to enhance viral infection, through the modification of the m6A levels on their corresponding mRNAs [192]. Moreover, virus-specific PTMs (such as dimethylcytosine species m5Cm and m44C) were only present in the ZIKV and HCV RNA genomes isolated from virions and enhanced viral replication though the recruitment of the nuclear DEAD-box containing RNA helicase DDX6 [193]. In addition, members of the IFIT family of antiviral RNA-binding proteins restrict infection by cytoplasmic RNA viruses through their ability to strongly bind 5′ capped non-self mRNAs (cap0) thereby preventing their translation. Alphaviruses antagonize IFIT1 function directly by inhibiting association with viral RNA through the generation of stable secondary structures in the 5′-UTR (untranslated region) [194]. The control of RNA PTM could therefore play a critical role in the ability of RNA viruses to escape innate antiviral responses.

The remarkable diversity of PTMs that have already been identified on both cellular [192] and viral RNAs [193] suggests not only very broad functional consequences, but also the likelihood that the high degree of modification of the PTM landscape induced by RNA viruses, illustrated by the Zika-induced alteration of m6A topology in host mRNAs, may have deleterious effects on a wide range of mechanisms involved in the development and control of cognitive function.

## Conclusions

Acute or chronic neuroinflammation is emerging as a key mechanism in various neuronal disorders [95, 195]. Viruses are now strongly considered as potential environment factors favoring the onset of brain diseases. For instance, viral “hit and run” occurring during pediatric Measles infection can be linked with the appearance of neuronal disorders few years after the initial infection [11]. In this context, arboviral infections may have long-lasting effects on the nervous system, as a result of the direct interaction of viruses with cells of the brain, or, indirectly, because of the neuroinflammatory status found associated with the infections. However, the mechanisms underlying potential viral persistence and the contribution of neuroinflammation to CNS pathophysiology are unclear. Notably, other environmental factors such as toxins and chemicals are now well linked to neurological diseases. Among these multifactorial causes, one has to consider the burden of arboviral infections, in particular in endemic regions where annual epidemics are occurring. Cohort studies are still highly needed, in particular to study long-term sequelae.

## Data Availability

Not applicable

## Abbreviations

AD: Alzheimer’s disease
AJ: Adherens junctions
BBB: Blood brain barrier
CCHMV: Crimean-Congo hemorrhagic fever virus
ChAT: Choline acetyl transferase
CHIKV: Chikungunya virus
CMV: Cytomegalovirus
CNS: Central nervous system
CP: Choroid plexus
CSF: Cerebrospinal fluid
CXCL10: Chemokine (C-X-C motif) ligand 10
CZS: Congenital Zika syndrome
DENV: Dengue virus
DNA: Deoxyribose nucleic acid
EEEV: Eastern Equine encephalitis virus
HAD: HIV-associated dementia
HAND: HIV-associated neurocognitive disorders
HCV: Hepatitis C virus
HIV: Human immunodeficiency virus
HSV-1: Herpes simplex virus-1
IFN: Interferon
IL: Interleukin
JEV: Japanese encephalitis virus
LACV: La Cross Virus
MAYV: Mayaro virus
m6A: N6-methyladenosine
MMP: Matrix metalloproteinase
mRNA: Messenger RNA
NK: Natural killer
NVU: Neurovascular unit
PNS: Peripheral nervous system
POWV: Powassan virus
PTM: Post-transcriptional modification
RVFV: Rift valley fever virus
RNA: Ribonucleic acid
TBEV: Tick-borne encephalitis virus
TJ: Tight junctions
TLR: Toll-like receptor
TNF-α: Tumor necrosis factors alpha
USUV: Usutu virus
VEEV: Venezuelan Equine encephalitis virus
WEEV: Western Equine encephalitis virus
WNV: West Nile virus
ZIKV: Zika virus
ZO-1: Zona occludens 1
